# The Physician’s Visiting List, Diary, and Book of Engagements, for 1861

**Published:** 1861-01

**Authors:** 


For Sale by S. C. Griggs & Co., Chicago.

---

THE PHYSICIAN'S VISITING LIST, DIARY, AND BOOK OF ENGAGEMENTS, for 1861. Published by Lindsay and Blakiston, Philadelphia.

This very useful pocket day-book, &c., &c., is too well known to the profession to require description or comment.

